# Knockdown hsa_circ_0063526 inhibits endometriosis progression via regulating the miR-141-5p / EMT axis and downregulating estrogen receptors

**DOI:** 10.18632/aging.203799

**Published:** 2021-12-30

**Authors:** Zhangming Wei, Yi Hu, Xiang He, Mengmeng Zhang, Xinyue Zhang, Yali Wang, Xiaoling Fang, Liping Li

**Affiliations:** 1 Department of Obstetrics, The Second Clinical Medical College, Jinan University, Shenzhen People's Hospital, Shenzhen 518020, Guangdong, China; 2 The First Affiliated Hospital, Jinan University, Guangzhou 510000, Guangdong, China; 3 Department of Obstetrics and Gynecology, The Second Xiangya Hospital, Central South University, Changsha 410000, Hunan, P.R. China; 4 The First Affiliated Hospital, Department of Obstetrics and Gynaecology, Hengyang Medical School, University of South China, Hengyang 421001, Hunan, China; 5 Xiangya Stomatological Hospital, Central South University, Changsha 410008, Hunan, China

**Keywords:** endometriosis, hsa_circ_0063526, EMT, circRNA, epigenetics

## Abstract

Endometriosis can cause severe social burdens. Abnormal circular RNA levels have been found to lead to changes of related gene expression, thereby mediating the occurrence and development of a series of diseases, including endometriosis. The role of circRNA in endometriosis is still in its infancy. This study will explore the role of circRNA hsa_circ_0063526 with microRNA-141-5p in the development of endometriosis. The expression levels of genes were detected by RT-qPCR. Transwell, wound-healing, and EdU assays were performed on the End1 / E6E7 cell line from the endometriosis patient. PCR and immunohistochemistry were used to detect the expression of candidate regulatory genes in ectopic lesions in an endometriosis mice model. The expression level of hsa_circ_0063526 in ectopic tissue of endometriosis patients was significantly higher than control (P<0.05), The expression levels of hsa_circ_0063526 and miRNA-141-5P in ectopic tissue of endometriosis were negatively correlated (P<0.05). Knockdown of hsa_circ_0063526 inhibited the invasion, migration, and proliferation ability of End1 / E6E7 cell; the inhibition of microRNA-141-5p rescued this inhibition (P <0.05). *In vivo* experiments showed that miR-141-5p and si-hsa_circ_0063526 treatment reduced lesion size and regulated endometriosis genes. Our data suggest that hsa_circ_0063526 and miR-141-5p are possible biomarkers and therapeutic targets for endometriosis.

## INTRODUCTION

Endometriosis is a common estrogen-dependent chronic disease affecting about 10% of childbearing-aged women [1]. The disease causes infertility and further malignant transformation, and 20-50% of infertile patients had endometriosis [2]. The etiology and pathogenesis of endometriosis are not clear. Sampson [3] first proposed the theory of menstrual blood reflux: the endometrial glandular epithelium and mesenchymal cells flow back into the fallopian tube and the abdominal cavity with menstrual blood, and implant in the bottom of abdominal organs such as the ovaries or pelvic peritoneum [4]. The cells continue to grow there, creating endometriosis [5]. Although endometriosis has benign histomorphology, its clinical behaviors show similar malignant tumors' invasion and resistance to apoptosis [5]. Recently, the pathogenesis of endometriosis has been increasingly attributed to a variety of genes and factors closely related to genetics [6]. Current treatment for endometriosis has many side effects, including stopping the menstrual cycle [7–10]. Because of this, new diagnostic indicators and non-hormonal therapy for endometriosis are urgently needed.

MicroRNAs (miRNA) are highly conserved and important post-transcriptional regulators [11]. At present, studies on the interaction between miRNA and endometriosis are gradually attracting attention. Many studies have regarded miRNA as a potential new biomarker for endometriosis [12–15].

Previous studies from our group used miRNA Solexa sequencing and RT-qPCR verification to test the serum samples of endometriosis patients in stage I and II [16–19]; the results showed that the expression level of miR-141 was lower than control [20]. Other studies have also demonstrated the down-regulation of the mir-200 family (including miR-141-5p) in plasma of endometriosis patients [20].

Several studies have explored the use of miR-141-5p to treat a range of diseases. For example, Kim et al. previously synthesized miR-141-5p hydrogel that demonstrated compelling results in liver cancer mice [21]. Rekker et al. reported that the expression of circulating microRNA-200s family, including miR-141-5p, in the serum of endometriosis patients was lower than control [14]. Liang et al. used miR-200c to suppress endometriosis *in vitro* and *in vivo* [22]. It has also been reported that the reduction of miR-141-5p in endometriosis tissue promotes the development of endometriosis mainly by regulating the epithelial-mesenchymal transformation (EMT) [23].

CircRNAs exist in eukaryotic cells as covalently closed rings, without 5 'or 3' polarity or polyadenylate, and are more conserved and stable than linear RNAs. [24] CircRNAs were previously thought to be a rare type of RNA, simply "waste products" from the process of RNA cutting [25]. However, increasing evidence has shown that circRNAs are involved in the proliferation, invasion, and metastasis of various diseases [26–29]. CircRNAs regulate gene expression levels mainly by binding to proteins or sponging microRNAs [30]. These molecular interactions will open up new prospects for diagnosing and treating endometriosis [23, 29, 31]. However, the pattern and potential role of circRNAs in the tissue of patients with endometriosis have not been elucidated.

**Figure 1:**
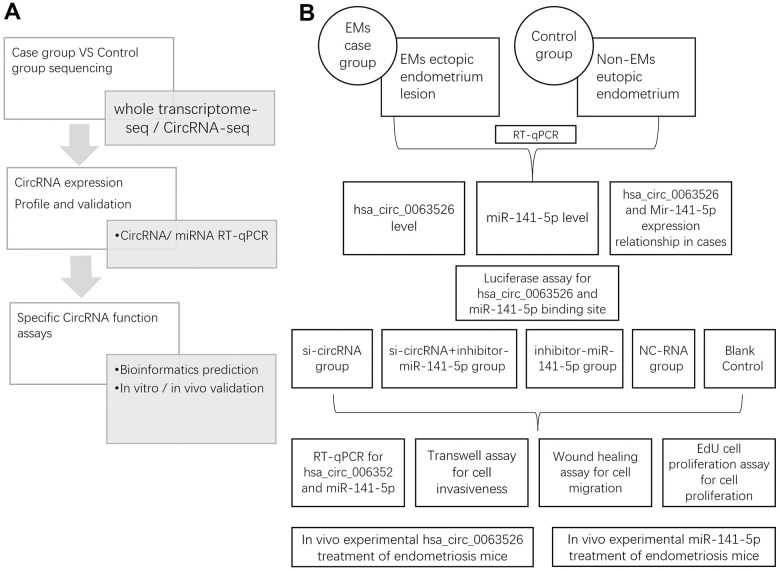
**Study design.** (**A**) Whole research design. sequencing, profile validation and function bioinformatics part has been done and published in our previous articles; (**B**) Experimental design in this study.

Our previous study tested microarray circRNA expression and performed RT-qPCR verification in four endometriosis tissue samples compared to four controls. We found that the expression level of hsa_circ_0063526 (circ-RanGAP1) was higher in the endometriosis group, and bioinformatics analysis revealed that circ-RanGAP1 and miRNA-141-5p have complementary binding sites [32]. We hypothesized that circRNAs might be involved in the development of endometriosis. Here, we aim to explore the correlation between hsa_circ_0063526 with miR-141-5p and endometriosis (Figure 1).

## RESULTS

### Comparison of relative expression of hsa_circ_0063526 between endometriosis patients and the control group

**Figure 2:**
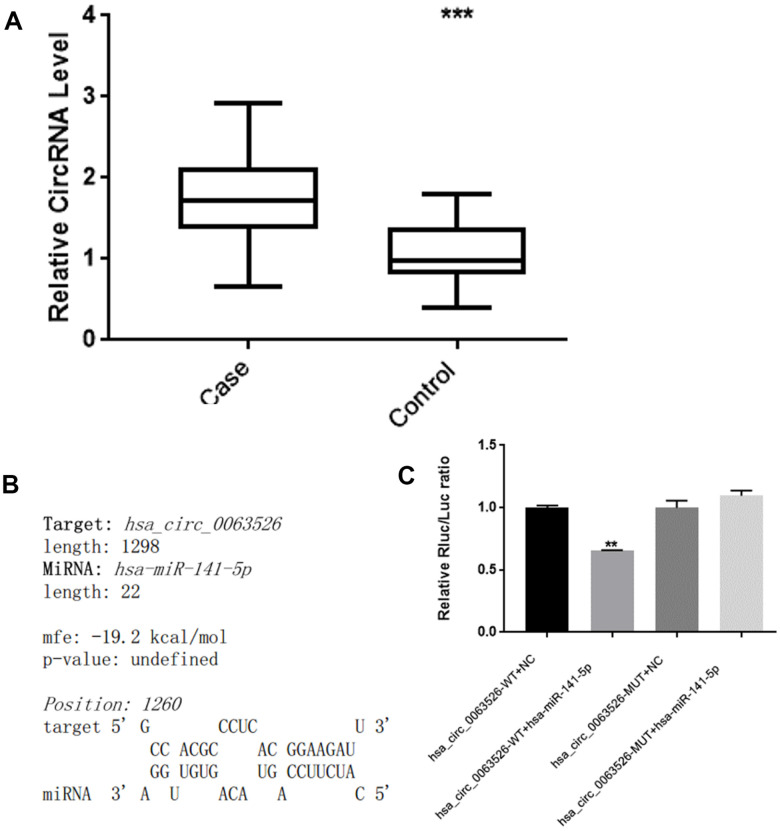
**hsa_circ_0063526 level is higher in EMs ectopic endometrial lesions than control’s eutopic endometrial tissue and hsa_circ_0063526 have binding site with miRNA-141-5p.** (**A**) RT-qPCR analysis of the hsa_circ_0063526 level in the endometriosis group and control group by RT-qPCR. n=41 (*** P<0.005). (**B**) hsa_circ_0063526 complementary sequence with miR-141-5p. (**C**) Luciferase reporter experiment showed that miR-141-5p can bind to hsa_circ_0063526 at this site. Replicated 3 times with 3 separate samples (** P<0.01).

RT-qPCR was used to analyze the expression level of hsa_circ_0063526 in the endometriosis group compared to the control group. The results are shown in Figure 2. Compared with the control group, the level of hsa_circ_0063526 was significantly higher in the endometriosis group; mean ± SEM was 0.686 ± 0.174 (P<0.005).

### Bioinformatics analysis and luciferase assay with hsa_circ_0063526 and miRNA-141-5p

We used bioinformatics analysis (starBase, www.starBase.sysu.edu.cn, RNAhybrid software) to predict the downstream target miRNA to hsa_circ_0063526 previously. We performed a double luciferase reporter assay on the binding site (Figure 2B, 2C). The double luciferase assay showed that the co-transfection of hsa_circ_0063526 wild-type and the miR-141-5p mimic significantly reduced luciferase activity compared with the control group. Compared with the co-transfection of hsa_circ_0063526 mutant and miR-141-5p mimic, the reporter gene expression was rescued, indicating that miR-141-5p mimic could bind to hsa_circ_0063526.

### Correlation between miR-141-5p and hsa_circ_0063526 expression

**Figure 3:**
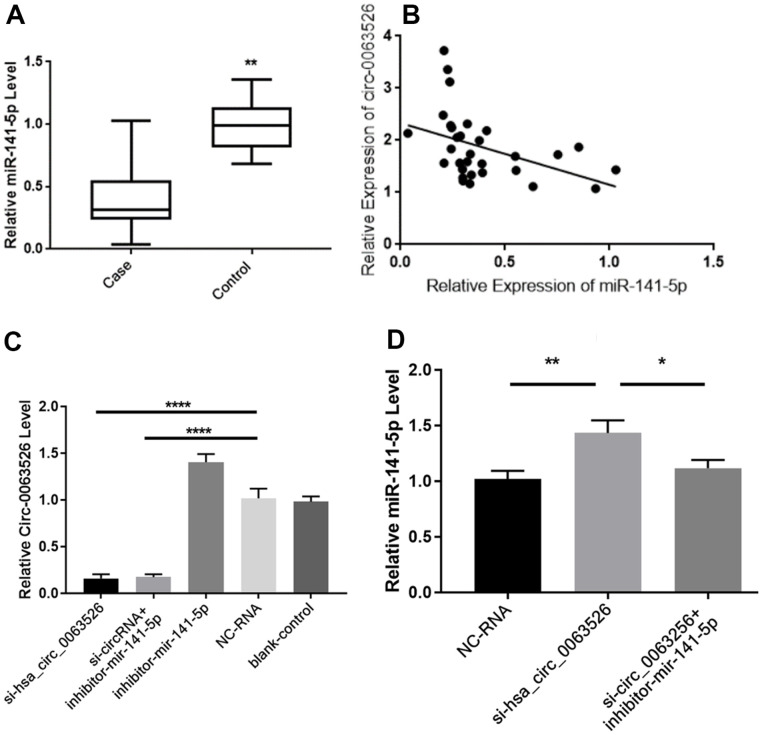
**Hsa_circ_0063526 and miR-141-5p expression level is negative correlated.** (**A**) Quantitative analysis of miR-141-5p expression levels in endometriosis group and control group by RT-qPCR.n=41 (** P<0.01). (**B**) Pearson’s correlation analysis of the correlation between hsa_circ_0063526 and miR-141-5p expression level in the endometriosis group. n=31(P<0.05) (**C**) RT-qPCR shows the relative expression level of hsa_circ_0063526 in End1/E6E7 cells after downregulation of hsa_circ0063526 or inhibition of miR-141-5p. (**** P<0.001). (**D**) RT-qPCR shows the relative expression level of miR-141-5p in End1/E6E7 cells after down-regulation of hsa_circ_0063526. (*(P<0.05, ** P<0.01). Replicated 3 times with 3 separate samples.

RT-qPCR was used to determine the relative expression of miR-141-5p between endometriosis patients and the control group (Figure 3A). We found a significant difference of means±SEM that was 0.600 ± 0.084 (P<0.01). Pearson’s correlation analysis of the expression levels of hsa_circ_0063526 and miR-141-5p in the lesion tissue of endometriosis showed a negative correlation (r= -0.427, p<0.05, Figure 3B). Pearson’s correlation analysis further indicated this negative interaction between hsa_circ_0063526 and miR-141-5p.

Due to the overexpression of hsa_circ_0063526, we constructed different siRNAs to knock down hsa_circ_0063526. Three fluorescent small interfering RNAs (siRNAs) were designed and synthesized. Fluorescent images showed successful transfection of siRNA into End1/E6E7 cells, and results showed that interference sequence three significantly reduced the expression of hsa_circ_0063526 compared to the other two (Supplementary Figure 1).

After transfection with si-hsa_circ_0063526, the expression level of hsa_circ_0063526 in End1/E6E7 cells in the si-hsa_circ_0063526 group was significantly decreased (P<0.05, Figure 3C), and the expression level of miR-141-5p was significantly increased (P<0.05, Figure 3D), compared with that in the blank group and the si-NC (Negative control) group. The results showed that hsa_circ_0063526 negatively regulated the expression level of miR-141-5p in End1/E6E7 cells.

### Down-regulation of hsa_circ_0063526 can inhibit the proliferation, invasion, and migration of End1 / E6E7 cells

**Figure 4:**
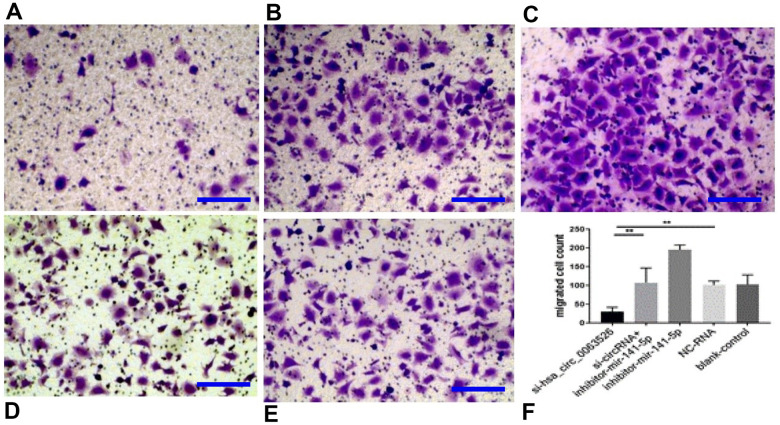
**Transwell assay to investigate the effect of inhibition of hsa_circ_0063526 or/and miR-141-5p on cell invasion of End1/E6E7 cells.** (**A**) si-hsa_circ_0063526 group; (**B**) si-hsa_circ_0063526 +inhibitor miR-141-5p group; (**C**) miR-141-5p Inhibitor group; (**D**) NC-RNA group; (**E**) Blank control group; (**F**) The number of cells comparing to the NC-RNA control group. after End1 / E6E7 cell transfected hsa_circ_0063526 siRNA, the cells entering the lower chamber through the pore was lesser compared to the control group, cell invasion ability decreased. After co-transfection with si-hsa_circ_0063526 + inhibitor miR-141-5p, the invasion ability of End1/E6E7 cells was rescued. Replicated 3 times with 3 separate samples (* P<0.05, ** P<0.01) (bar:25μm).

**Figure 5:**
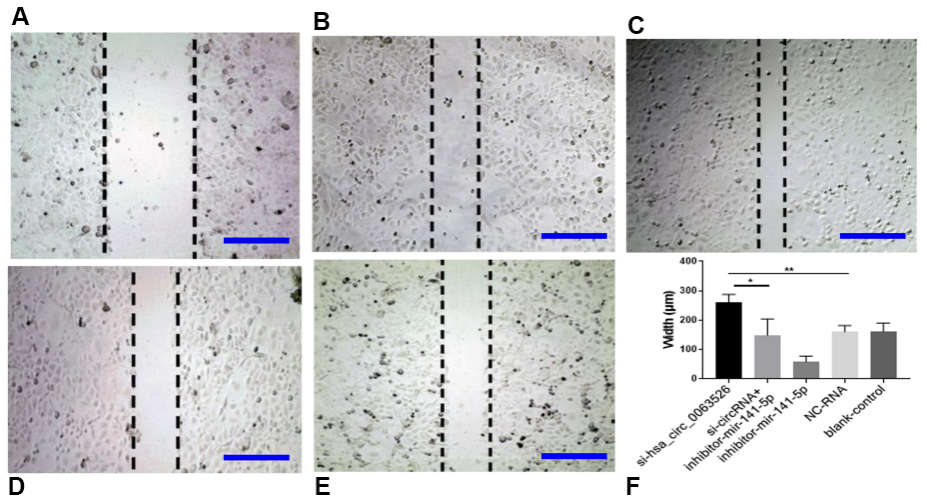
**Wound-healing test on the effect of inhibition of hsa_circ_0063526 or/and miR-141-5p on cell migration in End1/E6E7 cells.** (**A**) si-hsa_circ_0063526 group; (**B**) si-hsa_circ_0063526 +inhibitor miR-141-5p group; (**C**) miR-141-5p Inhibitor group; (**D**) NC-RNA group; (**E**) Blank control group; (**F**) The scratch width comparing to the si-NC group. After transfection with hsa_circ_0063526 siRNA in End1/E6E7 cells, the cell migration ability was decreased compared with the control group. After co-transfection with si-hsa_circ_0063526 + inhibitor miR-141-5p, the cell migration ability of End1/E6E7 cells was rescued. Replicated 3 times with 3 separate samples (* P<0.05, ** P<0.01) (bar:100μm).

Migration, invasion, and proliferation of ectopic endometrial cells are essential pathological processes for the development of endometriosis. Therefore, 72 hours after transfection of si-hsa_circ_0063526, this study further explored the changes of cell migration and invasion and cell proliferation ability after down-regulation of hsa_circ_0063526. After End1 / E6E7 cells were transfected with hsa_circ_0063526 siRNA, fewer cells entered the lower chamber than the control group, demonstrating that cell invasion ability decreased (Figure 4). The ability of cell migration was also tested by a wound healing experiment; after End1 / E6E7 cells were transfected hsa_circ_0063526 siRNA, the width of the cell scratch was broader than that of the control group, demonstrating decreased cell migration ability (Figure 5).

**Figure 6:**
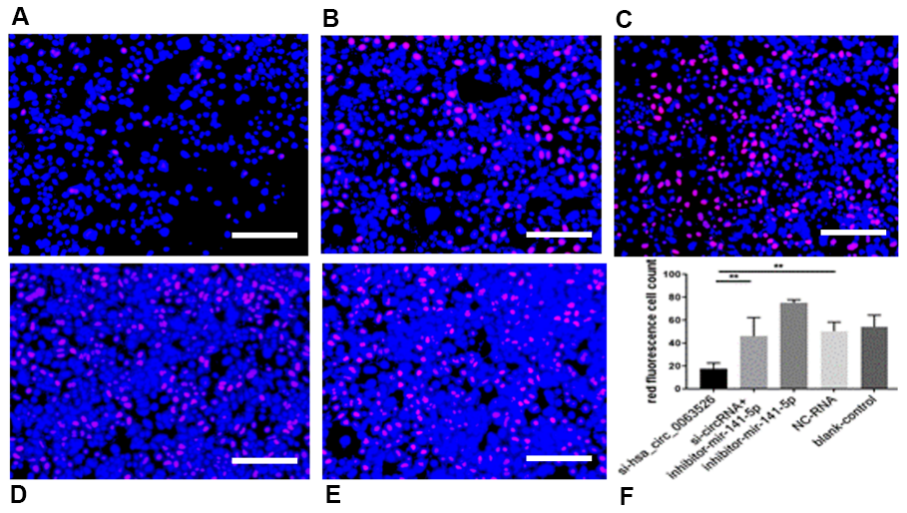
**EdU assay was used to investigate the effect of inhibition of hsa_circ_0063526 or/and miR-141-5p on the proliferation of End1/E6E7 cells.** (**A**) si-hsa_circ_0063526 group; (**B**) si-hsa_circ_0063526 +inhibitor miR-141-5p group; (**C**) miR-141-5p Inhibitor group; (**D**) NC-RNA group; (**E**) Blank control group; (**F**) the number of red fluorescent cells. After transfection with hsa_circ_0063526 siRNA in End1/E6E7 cells, the number of cells in the control group was lower, and the proliferation ability of cells in endometriosis was decreased. After co-transfection with si-hsa_circ_0063526 + inhibitor miR-141-5p, the proliferation ability of End1/E6E7 cells was rescued. Replicated 3 times with 3 separate samples (* P<0.05, ** P<0.01) (bar:25μm).

**Figure 7:**
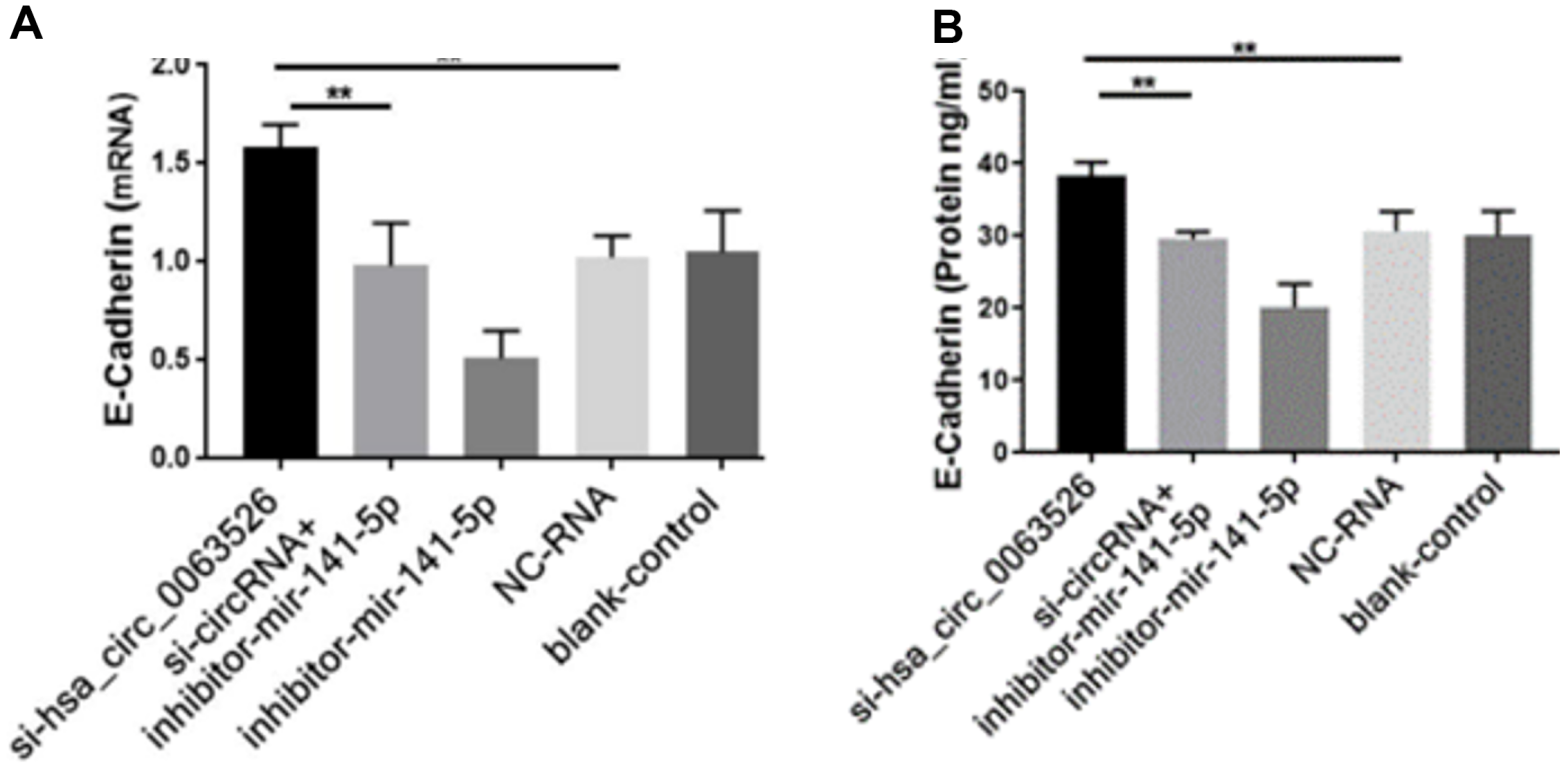
**RT-qPCR and ELISA assay to investigate the effect of inhibition of hsa_circ_0063526 or/and miR-141-5p on the expression of E-cadherin in End1/E6E7 cells.** (**A**) The expression level of E-cadherin mRNA (**B**) Expression level of E-cadherin protein. E-cadherin expression was upregulated. Expression changes were recovered after co-transfection with si-hsa_circ_0063526 + inhibitor- miR-141-5p. Replicated 3 times with 3 separate samples (* P<0.05, ** P<0.01).

Further, the effect of down-regulation of hsa_circ_0063526 on cell proliferation was detected using the EdU cell proliferation assay. By72 h after siRNA transfection, fewer End1/E6E7 cells were in the mitotic stage after the downregulation of hsa_circ_0063526, and the proliferation capacity of the cells was decreased after siRNA transfection compared to control. Difference between means± SEM=32.38 ± 6.639 (P<0.05, Figure 6). PCR and ELISA results showed that the expression of E-cadherin mRNA, an important epithelial marker of EMT, was upregulated (P<0.05, Figure 7).

### Downregulation of miR-141-5p can rescue the proliferation, invasion, and migration of endometriosis inhibited by hsa_circ_0063526

After cells were co-transfected with si-hsa_circ_0063526+inhibitor-miR-141-5p, the proliferation, invasion, and migration of endometriosis cells were rescued (P<0.05, Figures 4–6). PCR and ELISA results showed that the expression of E-cadherin, an essential epithelial marker of EMT, was downregulated compared with the si-hsa_circ_0063526 group (P<0.05, Figure 7).

The above experimental results showed that knockdown hsa_circ_0063526 might inhibit the development of endometriosis. Inhibition of microRNA-141-5p can rescue the inhibitory effect brought by knockdown of hsa_circ_0063526. Therefore, we hypothesize hsa_circ_0063526 promotes the development of endometriosis through inhibition of microRNA-141-5p.

### Comparison of miR-141-5p treatment and pathological changes

**Figure 8:**
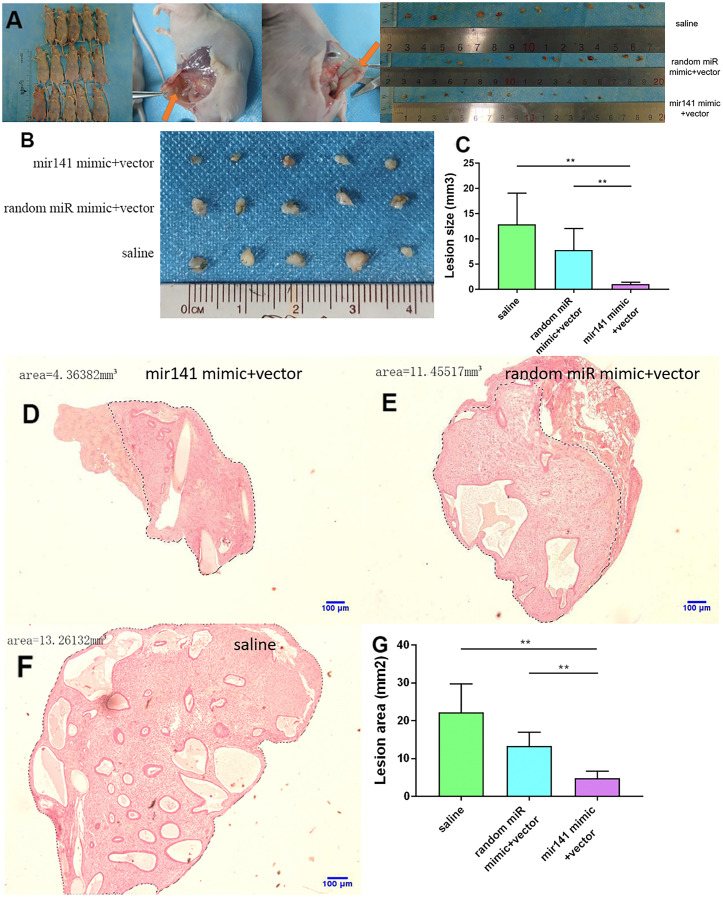
**Collection and comparison of lesion tissues from each mouse.** (**A**) The collection of endometriosis lesions. (**B**, **C**) Comparison of left abdominal lesion volume; (5 mice in each group). (**D**–**G**) The difference of histological area of the median section of endometriosis in mice of the upregulated group and control group under H&E staining (*P < 0.05, ** P < 0.01).

No adverse reactions were observed in mice treated with miR-141-5p. At the end of miRNA-141-5p treatment, mice were sacrificed by cervical dislocation and endometriosis lesions were collected and the volume of the lesions between the miR-141-5p treatment group and the control group were evaluated. All lesions were cystic and smallest in the miR-141-5p treated groups (Figure 8A–8C).

Further, the histological area of all lesions was observed under the microscope. The histological area of endometriosis in abdominal lesions of the miR-141-5p treatment group was significantly smaller than the two control groups (p < 0.05, Figure 8D–8G).

### Expression difference of endometriosis-related genes

**Figure 9:**
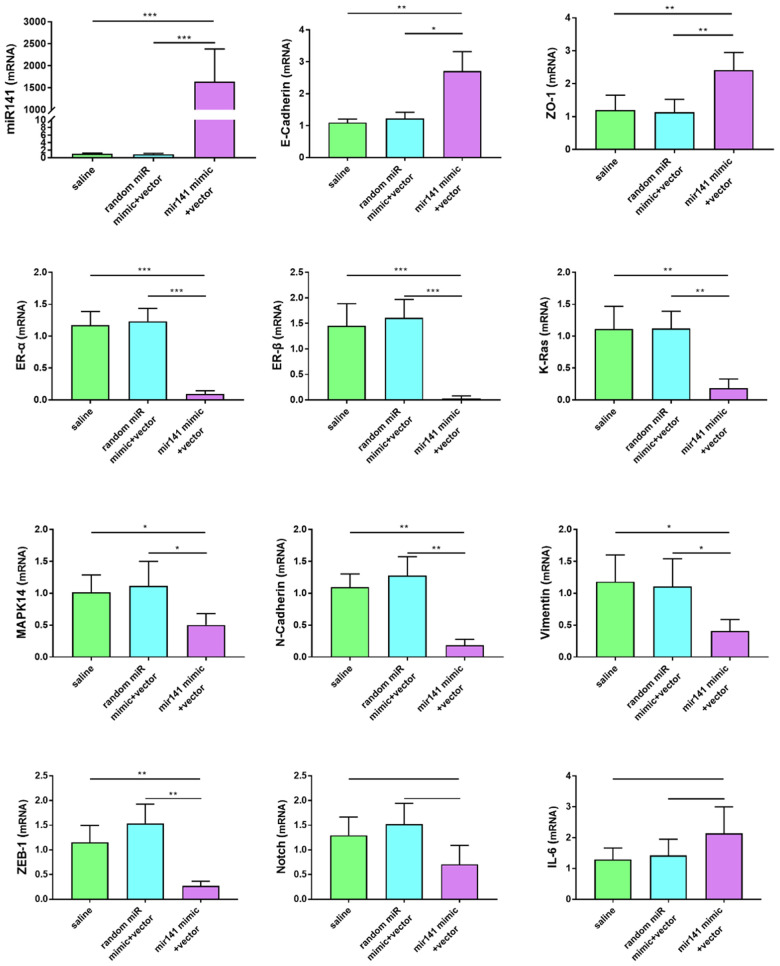
**Effects of miR-141-5p treatment on mRNA expression of pathologically related genes in endometriosis detected by RT-qPCR.** miR-141-5p treatment significantly decreased n-cadherin, Vimentin, K-ras, MAPK-14, ER-α, ER-β, ZEB-1 expression levels, and increased E-cadherin, ZO-1 expression levels. The data were shown as average percentage ±SEM. Replicated 3 times with 3 separate samples (*P < 0.05, ** P < 0.01).

The differences in gene expression levels related to endometriosis were detected by RT-qPCR. The expression levels of some genes known to promote the development of endometriosis decreased in the miR-141-5p group. Expression levels of N-cadherin, Vimentin, K-ras, MAPK-14, ER-α, ER-β, and ZEB-1 were decreased in the miR-141-5p treatment group (P<0.05, Figure 9). The decrease fold in gene expression is 6.5-fold for K-Ras, 1.81-fold for MAPK14, 13.9-fold for ER-α, 97.2-fold for ER-β, 4.63-fold for N-Cadherin, 2.37-fold for Vimentin, 3.98-fold for ZEB-1 in 141 treated group compared to saline group. Expression levels of miR-141, E-Cadherin, ZO-1 (Zona Occludens 1) were increased in the miR-141-5p treatment group (P<0.05, Figure 9). Expression levels of Notch and IL-6 were not statistically significant between treatment and control groups (P>0.05, Figure 9).

**Figure 10:**
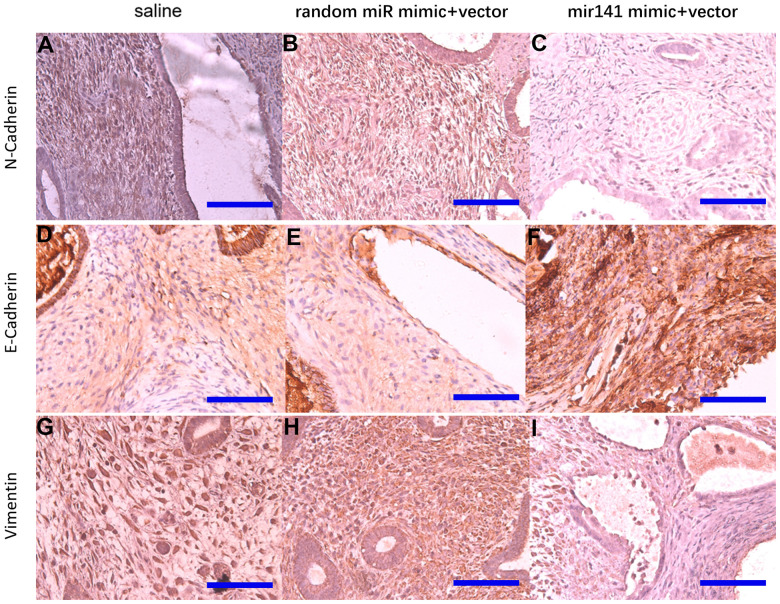
**Representative images of N-cadherin, E-cadherin, and Vimentin protein levels detected by immunohistochemistry.** N-cadherin protein (**A**–**C**), E-cadherin protein (**D**–**F**), Vimentin protein (**G**–**I**) (bar:50μm).

Immunohistochemistry results showed that N-cadherin and vimentin protein levels in stromal cells decreased in the miR-141-5p treatment group (Figure 10).

### Comparison of si-hsa_circ_0063526 treatment and pathological changes

**Figure 11:**
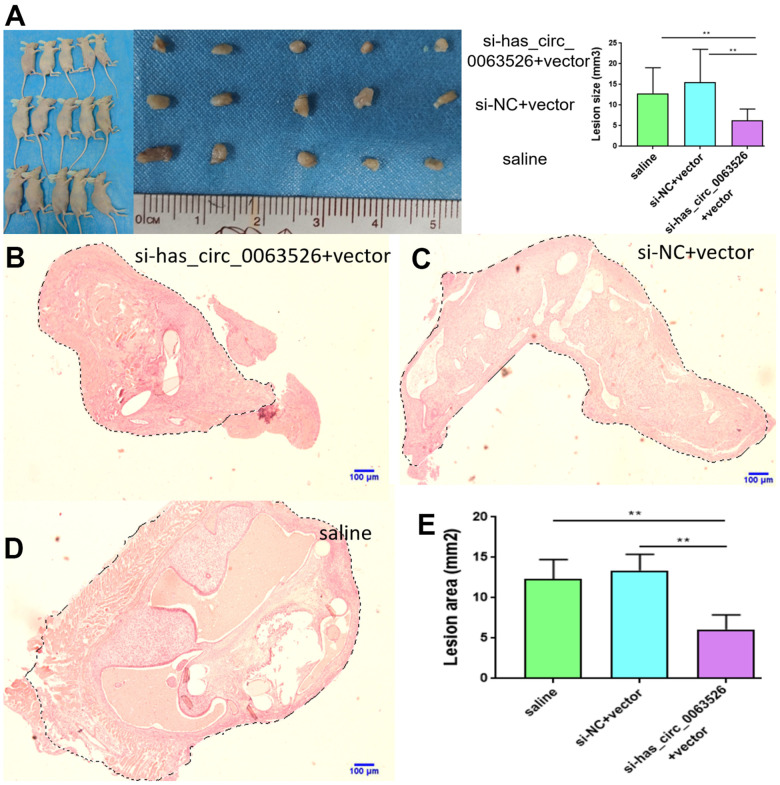
**Tissue collection and comparison of each mouse.** (**A**) Comparing the volume of lesions in the left abdomen; (5 mice in each group). (**B**–**E**) The difference of histological area of the median section of endometriosis in mice of the si-hsa_circ_0063526 treatment group and control group under H&E staining. Data are expressed as mean ±SEM, *P < 0.05, ** P < 0.01.

No adverse reactions were observed in mice treated with si-hsa_circ_0063526 agomir. At the end of si-hsa_circ_0063526 agomir treatment, mice were sacrificed by cervical dislocation and endometriosis lesions were collected. We evaluated the volume of the lesions between the si-hsa_circ_0063526 agomir treatment group and the control group. All lesions were cystic. Lesions in si-hsa_circ_0063526 agomir treatment groups were relatively small (Figure 11A).

Further, the histological area of all lesions was observed under the microscope, and the histological area of abdominal lesions in the miR-141-5p treatment group was significantly smaller than the two control groups (p < 0.05, Figure 11B–11E).

### Endometriosis-related gene expression differences

**Figure 12:**
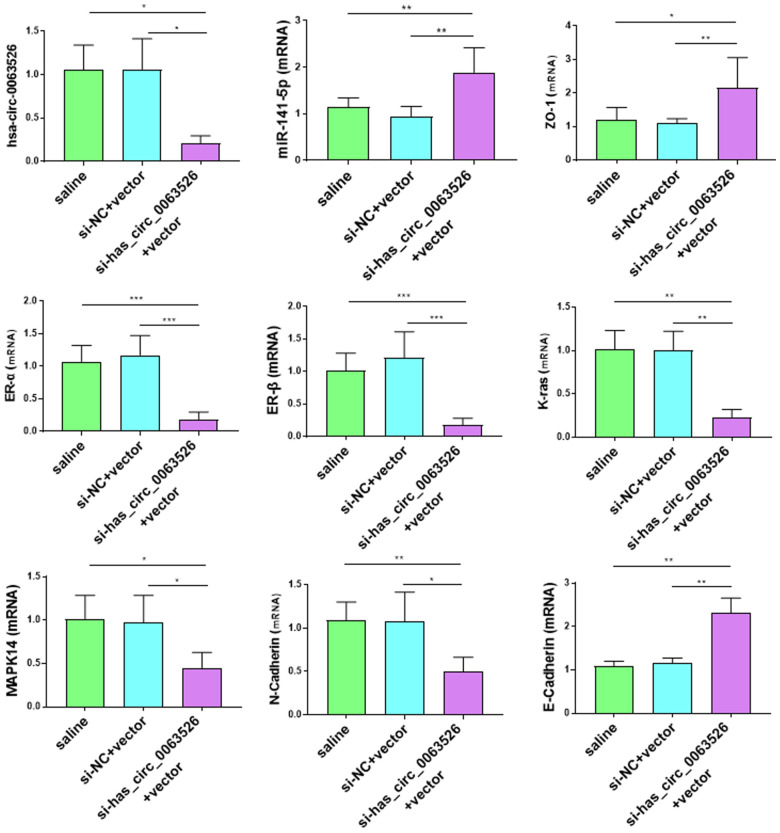
**Effects of si-hsa_circ_0063526 treatment on mRNA expression of pathologically related genes in endometriosis detected by RT-qPCR.** The expression levels of N-cadherin, K-ras, MAPK-14, ER-α, ER-β, in the si-hsa_circ_0063526 treatment group were significantly decreased, while the expression levels of E-cadherin and ZO-1 were increased. The data were shown as average percentage ±SEM. Replicated 3 times with 3 separate samples (*P < 0.05, ** P < 0.01).

RT-qPCR was used to detect the gene expression differences associated with endometriosis. Compared with the control group, some genes known to promote the development of endometriosis were decreased in the si-hsa_circ_0063526 agomir group, including MAPK-14, K-ras, N-cadherin, Vimentin, ER-α, ER-β (P < 0.05, Figure 12). K-ras, MAPK14, ER-α, ER-β, and N-cadherin decreased between 2-8 fold (Figure 12). The expression levels of E-cadherin and Zona occludens 1 (Zona Occludens 1) in the si-hsa_circ_0063526 agomir group were increased (P<0.05, Figure 12).

**Figure 13:**
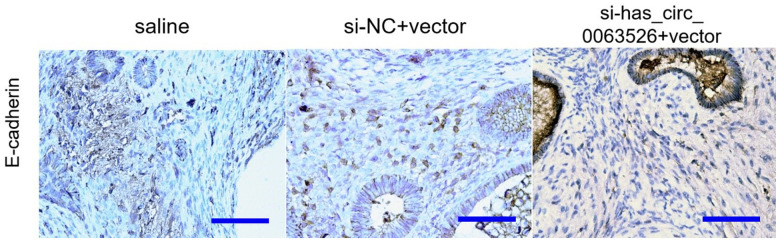
Representative images of E-cadherin protein levels detected by immunohistochemistry (bar:50μm).

Immunohistochemical results showed that E-cadherin staining intensity was significantly increased in the si-hsa_circ_0063526 treatment group (Figure 13).

## DISCUSSION

Endometriosis is a common estrogen-dependent chronic disease that affects about 10% women of childbearing age. While it is histomorphology is benign, its clinical behavior is similar malignant tumor invasion and resistance to apoptosis [6].

Searching for biomarkers for the detection and treatment of endometriosis has been an important area of research. The study of circRNA in endometriosis is still in its infancy, and its pattern and potential role in endometriosis tissues have not been elucidated [23, 33, 34]. Most of the studies on circRNA are preliminary, and more in-depth studies are needed to provide a sufficient basis for circRNA to be a diagnostic marker or therapeutic target for endometriosis [26, 29, 30, 35].

We verified that hsa_circ_0063526 was increased in endometriosis lesions, and the invasion, migration, and proliferation of End1/E6E7 cells were decreased after knockdown of hsa_circ_0063526. Bioinformatics has also shown that hsa_circ_0063526 may bind miR-141-5p as a target, and the dual-luciferase reporter assay further suggested that miR-141-5p can be combined with hsa_circ_0063526. In endometriosis lesions, the expression of both has a negative correlation, and our preliminary studies suggest that miR-141-5p regulates the development of endometriosis. Together, these data suggest that hsa_circ_0063526 may play a regulatory role in the occurrence and development of endometriosis through miR-141-5p.

Endometriosis is known for its cancer-like behavior [36]. It is characterized by aggressive invasion growth and a high risk of recurrence, similar to a malignant tumor [37, 38], therefore having similarities in pathogenesis. EMT endows cells with the ability to invade and metastasize, including reducing apoptosis, suppressing the immune response, and obtaining stem cell characteristics [39]. It not only plays a crucial role in embryonic development but is also involved in tissue healing, organ fibrosis, and the development of cancer [40]. Simpson's theory of menstrual blood reflux suggests that EMT is one of the drivers of endometriosis pathogenesis. After EMT, small mesothelial cells no longer provide a lamellar protective barrier between the basal layer and the lacunae. Because there is no protective barrier, endometrial cells tend to adhere to the peritoneal matrix, resulting in endometriosis. The expression of epithelial markers in endometriosis is down-regulated, and the expression of mesenchymal markers is upregulated [41]. EMT can resist apoptosis and assist the metastasis of ectopic lesions [42]. Second, the endometrium is formed by the transformation of the stromal cells during the development of the urogenital system of the embryo. Due to the retention of some stromal origin imprints, endometrial epithelial cells tended to revert to the original stromal state through EMT [43, 44]. We found that the EMT activity in the si-hsa_circ_0063526 group was decreased, and the expression level of E-cadherin was increased.

The Ras/MAPK signaling pathway is essential in embryo development, differentiation, proliferation, cell death, and other critical cellular processes [45]. K-ras activation is a classical method to establish a mouse model of spontaneous endometriosis and is upregulated [36, 46]. Here, we found that the expression level of K-ras was lower in the miR-141-5p and si-hsa_circ_0063526 transfected groups compared to the control.

Compared with linear RNA, circRNAs have a more stable structure and can resist the degradation of multiple RNA enzymes. Therefore, circRNAs could be a diagnostic and therapeutic target for endometriosis [47, 48]. Currently, no studies have been reported on the role of hsa_circ_0063526 in endometriosis, but other circRNA studies have shown that circRNA has potential value in prognosis prediction or early diagnosis of endometriosis.

Endometriosis is considered an estrogen-dependent disease, and ER-α&β have essential roles in endometriosis [42]. ER-α&β expression was significantly inhibited with miR-141-5p and si-hsa_circ_0063526, suggesting that si-hsa_circ_0063526/miR-141-5p pathway blocks estrogen stimulation in the treatment. These data reveal that miR-141-5p and si-hsa_circ_0063526 therapy may specifically block the sex hormone pathway in patients with endometriosis without systemic side effects of estrogen deficiency.

In this study, miR-141-5p and si-hsa_circ_0063526 were used for local therapy. After systematic administration, oligonucleotides have difficulty reaching the desired tissue because they are degraded by the liver and removed from the blood [49]. Therefore, many vectors have been used to improve the stability of oligonucleotides; however, these drugs are difficult to achieve targeted drug delivery [21]. We posit that miR-141-5p and si-hsa_circ_0063526 have therapeutic effects as an intraperitoneal injection [50]. However, since animal models cannot wholly simulate the patient's condition, the model could not reflect the role of inflammation, angiogenesis, and other activities in endometriosis, and the side effects of the therapy could not be observed as thoroughly as in the patient. Before being used in humans, dose-response and safety studies must be conducted.

In summary, miR-141-5p and si-hsa_circ_0063526 therapy reduce endometriosis development. The pleiotropic nature of miR-141-5p and si-hsa_circ_0063526 therapy suggests that multiple complementary mechanisms play a role in endometriosis. These effects suggest that endometriosis may be treated more comprehensively without the systemic side effects of current drugs, but further dose-response and safety studies are needed before they can be used in humans.

## MATERIALS AND METHODS

### Materials

### Clinical specimens of endometrial tissue

Tissue samples were collected from child-bearing-aged women who underwent surgery from February 2019 to January 2020 in the gynecology department of The Second Xiangya Hospital. Fresh proliferation-stage endometriosis cyst samples and endometrial tissue were snap-frozen and stored in a liquid nitrogen container. The study was approved by the ethics committee of The Second Xiangya Hospital. Before the samples were taken, informed consent was assigned by each patient. The clinicopathological data of endometriosis patients, including age, histological subtype, clinical-stage, and cyst size, was retrieved (Supplementary Table 1).

Experimental group: A total of 31 patients with ectopic endometriosis were enrolled, all of whom were diagnosed with ovarian endometriosis by laparoscopy and histopathology. The endometriosis lesions were collected intraoperatively as the ectopic endometrium group (American Fertility Society stage III-IV).

Control group: 10 patients with secondary infertility caused by fallopian tube obstruction factors confirmed by the combined operation of uterus and abdomen. The histological diagnosis is proliferation stage endometrium. The sample size was calculated with equation N=Z ^2^ ×(P ×(1-P))/E ^2^ and based on previous studies [51].

### Endometrial and renal cells lines

Human renal epithelial HEK293T cells and End1/E6E7 endometrial cells from endometriosis patients were purchased from Bena Culture Collection (Beijing, China).

### Plasmids for luciferase assay

pmiR-RB-Report™ hsa_circ_0063526 (hsa_circ_0063526 3’UTR:1044-1103) –WT (hsa_circ_0063526-WT), pmiR-RB-Report™ hsa_circ_0063526 (hsa_circ_00635263’UTR:1044-1103) - MUT (1273-1280GGAAGAT>CCTTCTA) (hsa_circ_0063526-MUT) plasmids were provided by Ribobio (Guangzhou, China).

### Primers used for RT-qPCR detection

All primers were provided by Ribobio (Guangzhou, China) and verified by NIH BLAST (Bethesda, MD). The primers used in the experiment are shown in Table 1.

**Table 1:** Primer information used in this paper.

**Primers**	**Sequences of 5 'to 3'**
ZO-1 Forward	GAATGATGGTTGGTATGGTGCG
ZO-1 Reverse	TCAGAAGTGTGTCTACTGTCCG
E-cadherin Forward	TCCATTTCTTGGTCTACGCC
E-cadherin Reverse	CACCTTCAGCCAACCTGTTT
N-cadherin Forward	GTGCCATTAGCCAAGGGAATTCAGC
N-cadherin Reverse	GCGTTCCTGTTCCACTCATAGGAGG
Vimentin Forward	AGCCGAAAACACCCTGCAAT
Vimentin Reverse	CGTTCAAGGTCAAGACGTGC
K-ras Forward	AGACACAAAACAGGCTCAGGA
K-ras Reverse	TTCACACAGCCAGGAGTCTTT
Beta-actin Forward	GGGGTGTTGAAGGTCTCAAA
Beta-actin Reverse	GGCATCCTCACCCTGAAGTA
ER-alpha Forward	AAGAGCTGCCAGGCCTGCC
ER-alpha Reverse	TTGGCAGCTCTCATGTCTCC
ER-beta Forward	GCTCAATTCCAGTATGTACC
ER-beta Reverse	GGACCACATTTTTGCACT
IL-6 Forward	GTCAACTCCATCTGCCCTTCAG
IL-6 Reverse	GGTCTGTTGTGGGTGGTATCCT
ZEB1 Forward	TGAATCATCGCTACTCCTACTGT
ZEB1 Reverse	TTTCACTGTCTTCATCCTCTTCCC
Notch-1 Forward	GTCAACGCCGTAGATGACC
Notch-1 Reverse	GTCAACGCCGTAGATGACC
MAPK14 Forward	GAACAAGACAATCTGGGAGGTG
MAPK14 Reverse	TTCGCATGAATGATGGACTGAA
GAPDH Forward	GCACCGTCAAGGCTGAGAAC
GAPDH Reverse	TGGTGAAGACGCCAGTGGA
hsa_circ_0063526 Forward	AGATTCTGGACCCTAACACTGG
hsa_circ_0063526 Reverse	CTCTTGCCTTTGAAACTCAGCT
miR-141-5p Forward	CGCGCATCTTCCAGTACAGT
miR-141-5p Reverse	AGTGCAGGGTCCGAGGTATT
miR - 141-5p reverse transcription	GTCGTATCCAGTGCAGGGTCCGAGGTA- TTCGCACTGGATACGACTCCAAC

### Design and synthesis of si-hsa_circ_0063526 and miR-141-5p inhibitor

For the sequence of the target gene hsa_circ_0063526, siRNA sequence and random independent short RNA sequence (NC-RNA) as the control were designed and synthesized by Genepharma (Suzhou, China): si-hsa_circ_0063526-1 Sense: 5 '- GacccuaacacugggucugTT-3' Antisense: 5 '- cagAcccaguguuagGGUCTT-3' si-hsa_circ_0063526-2 Sense: 5 '- AacacugggucugCagauctt-3' Antisense: 5 '- GaucugcagAcccaguguutt-3' si-hsa_circ_0063526-3 Sense: 5 '-cacugggucugCagaucuctt-3' Antisense: 5 '-GagaucugcagACCcagugTT-3' Inhibitor-miR-141-5p: a short-stranded RNA modified by the complementary sequence of miR-141-5p, provided by Ribobio(Guangzhou, China). MicroRNA-141-5p sequence is as follows: microRNA-141-5p: 5'-UAacaccugucugGuaaagaugg-3'.

### RNA extraction and RT-qPCR

RNA was extracted by Trizol reagent (Beijing Dingguo Biological Technology Co., Ltd.). Total RNA was reverse transcribed into cDNA, and real-time-qPCR analysis was performed using Universal SYBR Green MasterMix Kit (Vazyme, China). The 2^-∆∆Ct^ method was used for calculating the relative RNA expression levels [52].

### Bioinformatics analysis of hsa_circ_0063526 complementary miRNAs

Miranda, TargetScan, RNA22 v2, and RNAhybrid software were used to predict the potential target miRNAs and the target genes of the miRNAs involved. The complementary miRNA to hsa_circ_0063526 was predicted, and further research and verification were conducted.

### Dual-luciferase reporter assays for circRNA and miRNA binding

The recombinant plasmid of double luciferase reporter hsa_circ_0063526-WT and hsa_circ_0063526-MUT was designed and synthesized according to the complementary pairing sequence of miR-141-5p and hsa_circ_0063526-MUT. 293T cells were seeded with 1.0×10^4^ cells per well. The miRNA mimics or non-target control vector and target gene 3 'UTR double reporter vector or mutant vector were diluted in 5μL Opti-MEM medium, and the transfection reagent was diluted with 0.25μL Lipo6000 in 5μL Opti-MEm medium. Before plasmids and mimics were added to the cells, 90μL culture medium was added to each well. Mimics transfection concentration was 50nM, and plasmid concentration was 50ng/well. Each group included three replicates. After 48h of transfection, the medium was extracted and PBS added. Luciferase reagent was added and shaken for 10min. The medium was transferred to LUMITRAC™ 200 96 well white cell culture plate for fluorescence determination. Finally, 30μL stop reagent was added to each well for spectrophotometric determination.

In section 2.5-2.8, End1/E6E7 cells were divided into five groups, specifically:

1) si-hsa_circ_0063526 group (si-hsa_circ_0063526 transfection group): The si-hsa_circ_0063526 siRNA was transfected to knockdown hsa_circ_0063526.

2) si-hsa_circ_0063526 + inhibitor-miR-141-5p group (si-hsa_circ_0063526 + inhibitor-miR-141-5p group): the si-hsa_circ_0063526 and miR-141-5p were transfected, knockdown of hsa_circ_0063526 siRNA and inhibition of miRNA-141-5p performed at the same time.

3) Inhibitor of miR-141-5p group (inhibitor of miR-141-5p transfection group): Transfected the inhibitor of miR-141-5p to inhibit the function of miR-141-5p.

4) NC-RNA group (NC-RNA transfection group): group transfected with random short RNA sequences.

5) Blank control group: transfection reagent and RNA were not added.

### Wound healing assays for cell migration

To detect the migration of cells, we conducted wound healing assays *in vitro.* Sterile 100μL tips were dragged over the confluent monolayer cells in each cell culture wells to create a gap in the monolayer. Cells were washed with PBS and placed back into the incubator for 48 hours. The cells were observed under an inverted microscope. The width of the scratch was measured and recorded by ImageJ software (NIH, USA).

### Transwell invasion assays for cell invasiveness

To detect the invasion ability of each group, a Transwell assay was performed *in vitro.* Matrigel was transferred from -20° C to 4° C and thawed for 12 hours. The Matrigel was diluted without serum. The Matrigel was carefully placed into an upper chamber with a diameter of 8μm. The Transwell plate was placed back into the CO_2_ incubator at 37° C and incubated for 24 hours. 1×10^4^ of End1/E6E7 cells in 200μL serum-free cell suspension was added to the upper chamber. 600μL high-sugar DMEM medium containing 10% fetal bovine serum was added into the lower chamber, and the plate was put back into CO_2_ at 37° C and incubated for 36 hours. The cells were stained with 0.5% crystal violet and migrated cells were counted with ImageJ software.

### EdU assays for cell proliferation

EdU (5-acetylene 2' -deoxyuridine) proliferation assay was performed using the EdU Apollo567 proliferation *in vitro* kit (RiboBio, Guangzhou, China).

End1/E6E7 cells (2×10^4^) were seeded to a 96-well plate. The cells were transfected with si-hsa_circ_0063526, miR-141-5p inhibitor, si-circ_0063526 + miR-141-5p inhibitor, and control siRNA. After 48 hours, 100 μL diluted 50 M EdU was added to each 96-well plate. The cells were then fixed and dyed with 1X Apollo staining solution and DAPI. The number of EdU positive cells and the total number of cells were calculated immediately, and results were analyzed with ImageJ software.

### Enzyme-linked immunosorbent assay (ELISA) for E-cadherin

Human E-Cadherin ELISA kit (R&D Systems, MN, USA) was used for E-Cadherin expression detection. 100μL cell culture supernatants of the five groups were first added to the wells of the 96-well ELISA plate, along with a standard curve. The standard curve was set up in concentrations of 0, 0.31, 0.63, 1.25, 2.50, 5.00, 10.00, 20.00 ng/ml. The enzyme-labeled plates were incubated on a micro-oscillator with a speed of 700rpm for 2 h and washed 4 times. Next, 200μL of the enzyme marker E-cadherin conjugate was added into each well. Finally, substrate solution and termination solution were added to each well and the OD value at 450 nm was measured. Using the OD value of E-cadherin standard as ordinate (taking the average value of the concentration of three pores) and dilution concentration of a standard substance as abscissa, the standard curve was drawn, and the E-cadherin contents of the samples were calculated according to the standard curve.

### Induction of endometriosis in mice

The endometrium was obtained from 18 childbearing-aged women who underwent hysterectomy for uterine myoma at The Second Xiangya Hospital. Pelvic cavities were examined for the absence of endometriosis during the surgery. According to Noyes et al. criteria (1975), the menstrual cycle has been histologically confirmed as the proliferative phase. The patients had not received hormone therapy for at least six months. All experimental protocols were approved by The Ethics Committee under the declaration of Helsinki, and all women signed informed consent. The endometrium was obtained by an endometrial specimen collector (J-ES-090500; Cook, USA), cut into identical 2 mm diameter fragments, and incubated in DMEM, PEN/STREP (Changsha, China) culture medium at 4° C before implantation. Female nude mice aged 5 to 6 weeks were purchased and reared in the Department of Laboratory Animals in Central South University. Five animals were maintained in each cage during a 12-hour light and 12-hour dark cycle (8 a.m. to 8 p.m.) in a barrier system.

The modified endometriosis model previously used in our laboratory and widely by previous scholars was used to induce endometriosis in 30 mice [51]. In this protocol, endometrial tissue fragments of patients of the same size (2mm) were sutured to the peritoneal surface of each mice. Mice were anesthetized by sodium pentobarbital and laparotomies were performed through a midline incision. One piece of endometrial tissue fragments was sutured by 5-0 polyglactin suture on the surface of the left and right abdominal wall respectively before the peritoneum and skin were closed.

### microRNA-141-5p-agomir and si-hsa_circ_0063526 agomir treatment

Thirty experimental endometriosis models were randomly allocated into six groups of 5 in each. Five days after induction of endometriosis, miRNA-141-5p therapy began with miRNA-141-5p-agomir (uaacacugucugguaaagaugg Genepharma Company, China) +vector Entranster-R4000 carrier (Engreen Biosystem, China), or scramble miRNA-agomir +vector, or only saline as two control groups. si-hsa_circ_0063526 agomir therapy began with siRNA-hsa_circ_0063526 agomir (sense: 5 '-CACUGGGUCUGCAGAUCUCTT-3' Antisense: 5'-GAGAUCUGCAGACCCAGUGTT-3' Genepharma Company, China) +vector or scramble agomir+vector or only saline as two control group. RNAs were intraperitoneally injected into the mice by transfection vector Entranster-R4000 carrier. An Entranster-R4000+RNA mimic mixture was prepared. Each injection of 0.5mL 5% dextrose mixture included 90μg oligonucleotides and 20μL of transfection vector. The mice were injected intraperitoneally every three days for two weeks.

### Evaluation of lesions’ volume

After microRNA-141-5p-agomir and si-hsa_circ_0063526-agomir treatment for two weeks, the mice were sacrificed, and the endometriosis lesions were collected from the abdominal cavity of mice. The volume of lesions was calculated by the formula of minimum diameter^2^ * maximum diameter/2 [51]. Left abdominal lesions were preserved in RNA stabilized solution (Qiagen, Germany) for extraction of mRNA, RT-qPCR was used to detect gene expression, and the right abdominal lesions were preserved in 4% polyformaldehyde solution for immunohistochemical study. Endometriosis was observed under a light microscope after H & E staining in a blinded manner. Image J was used for the calculation of the lesion area. We import the scale bar in Image J. Then use Selection Brush Tool to calculate the total area of endometrial and stroma part.

### Immunohistochemistry for epithelial and mesenchymal markers

We used 4% paraformaldehyde to fix lesions and paraffin to embed lesions. Tissue was cut into slices about 5μm thick and fixed on glass slides, then boiled in sodium citrate (pH=6) solution in high pressure for 15 minutes for antigen retrieval. 10% goat serum was used for antigen blocking. Slides were incubated at 4° C overnight with anti-E-cadherin (1:1500; Proteintech, USA), anti-N-cadherin (1:1500; Proteintech, USA), anti-Vimentin (1:1500; Proteintech, USA) antibodies to determine protein expression. Sections were dyed with DAB (Well-Biology, Changsha, China) and counterstained with hematoxylin (Well-Biology, Changsha, China). Stained section images were taken with OLYMPUS BX63 (OLYMPUS, Japan) and analyzed by Image-pro Plus 7.0.

### Statistical analysis

All statistical analysis was performed in GraphPad Prism software (GraphPad, La Jolla, CA), and all *in vitro* experiments were carried out three times. First, normality and homogeneity of variance were tested in the data obtained from each group. If the sample mean of the group conformed to the homogeneity of variance and normality after comparison, one-way ANOVA was used, and the multiple comparisons between pairs was tested by the least significant difference (LSD) method. If the variance of the group of data is not uniform, the non-parametric Kruskal-Wallis H test and U test were used. Pearson’s correlation analysis was used for correlation analysis of numerical variables when two variables are in line with normal distribution, and Spearman correlation analysis was used if the two variables were not normally distributed. The test criterion was set to α=0.05.

### Availability of data and materials

All data are available to others with investigator support.

## Supplementary Material

Supplementary Figure 1

Supplementary Table 1
