# Supplementary material for: Knockdown hsa_circ_0063526 inhibits endometriosis progression via regulating the miR-141-5p / EMT axis and downregulating estrogen receptors
Source: Aging (Albany NY). 2021 Dec 30;13(24):26095–117. doi: 10.18632/aging.203799 (PMC8751610; doi:10.18632/aging.203799)
Supplement: Supplementary Figure 1 [file aging-13-203799-s001.pdf]

SUPPLEMENTARY FIGURE

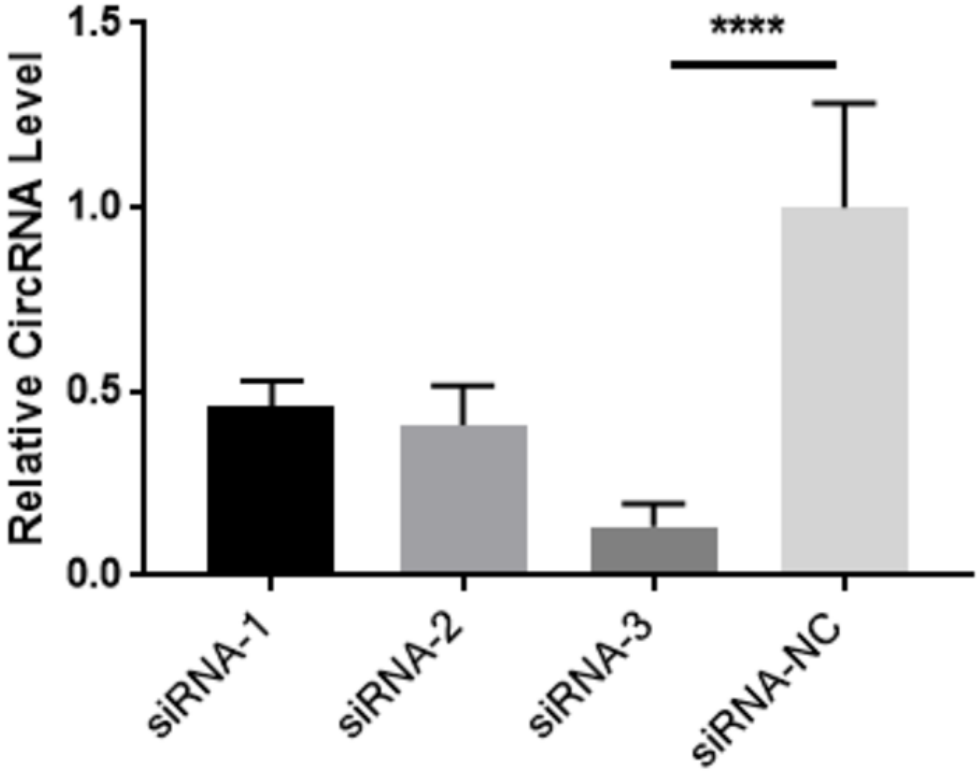

Supplementary Figure 1. Relative expression level of hsa_circ0063526 in End1/E6E7 cells after siRNA transfection. Replicated 3 times involving 3 samples (**** P<0.001).
