# Supplementary material for: Knockdown hsa_circ_0063526 inhibits endometriosis progression via regulating the miR-141-5p / EMT axis and downregulating estrogen receptors
Source: Aging (Albany NY). 2021 Dec 30;13(24):26095–117. doi: 10.18632/aging.203799 (PMC8751610; doi:10.18632/aging.203799)
Supplement: Supplementary Table 1 [file aging-13-203799-s002.pdf]

SUPPLEMENTARY TABLE

Supplementary Table 1. Clinical characteristics of the patients.

Specimen number	Diseases	The menstrual cycle	Source of parts	Age	Stage
YW-B-01	Ovarian endometriosis	Proliferative phase	The right ovary	28	IV
YW-B-02	Ovarian endometriosis	Proliferative phase	The right ovary	30	III
YW-B-03	Ovarian endometriosis	Proliferative phase	The right ovary	41	III
YW-B-04	Ovarian endometriosis	Proliferative phase	The right ovary	26	III
YW-B-05	Ovarian endometriosis	Proliferative phase	The right ovary	26	III
YW-B-06	Ovarian endometriosis	Proliferative phase	The left ovary	31	IV
YW-B-07	Ovarian endometriosis	Proliferative phase	The left ovary	28	IV
YW-B-08	Ovarian endometriosis	Proliferative phase	The left ovary	28	IV
YW-B-09	Ovarian endometriosis	Proliferative phase	The right ovary	38	III
YW-B-10	Ovarian endometriosis	Proliferative phase	The right ovary	24	III
YW-B-11	Ovarian endometriosis	Proliferative phase	The right ovary	28	III
YW-B-12	Ovarian endometriosis	Proliferative phase	The right ovary	29	III
YW-B-13	Ovarian endometriosis	Proliferative phase	The right ovary	26	III
YW-B-14	Ovarian endometriosis	Proliferative phase	The left ovary	38	IV
YW-B-15	Ovarian endometriosis	Proliferative phase	The right ovary	34	III
YW-B-16	Ovarian endometriosis	Proliferative phase	The left ovary	35	IV
YW-B-17	Ovarian endometriosis	Proliferative phase	The left ovary	31	IV
YW-B-18	Ovarian endometriosis	Proliferative phase	The left ovary	29	III
YW-B-19	Ovarian endometriosis	Proliferative phase	The right ovary	27	III
YW-B-20	Ovarian endometriosis	Proliferative phase	The right ovary	38	IV
YW-B-21	Ovarian endometriosis	Proliferative phase	Bilateral ovaries	26	IV
YW-B-22	Ovarian endometriosis	Proliferative phase	The left ovary	35	IV
YW-B-23	Ovarian endometriosis	Proliferative phase	The left ovary	30	III
YW-B-24	Ovarian endometriosis	Proliferative phase	The right ovary	38	IV
YW-B-25	Ovarian endometriosis	Proliferative phase	The right ovary	38	III
YW-B-26	Ovarian endometriosis	Proliferative phase	The right ovary	29	III
YW-B-27	Ovarian endometriosis	Proliferative phase	The right ovary	31	III
YW-B-28	Ovarian endometriosis	Proliferative phase	The right ovary	29	III
YW-B-29	Ovarian endometriosis	Proliferative phase	The right ovary	38	III
YW-B-30	Ovarian endometriosis	Proliferative phase	The right ovary	27	III
YW-B-31	Ovarian endometriosis	Proliferative phase	The right ovary	26	IV
DZ-N-01	Secondary infertility	Proliferative phase	Endometrium	40	-
DZ-N-02	Secondary infertility	Proliferative phase	Endometrium	41	-
DZ-N-03	Secondary infertility	Proliferative phase	Endometrium	32	-
DZ-N-04	Secondary infertility	Proliferative phase	Endometrium	30	-
DZ-N-05	Secondary infertility	Proliferative phase	Endometrium	27	-
DZ-N-06	Secondary infertility	Proliferative phase	Endometrium	26	-
DZ-N-07	Secondary infertility	Proliferative phase	Endometrium	39	-
DZ-N-08	Secondary infertility	Proliferative phase	Endometrium	29	-
DZ-N-09	Secondary infertility	Proliferative phase	Endometrium	43	-
DZ-N-10	Secondary infertility	Proliferative phase	Endometrium	27	-
